# Real-world data-based assessment of therapy-related myeloid neoplasms after poly(ADP-ribose) polymerase inhibitor treatment in ovarian cancer

**DOI:** 10.3389/fonc.2026.1728766

**Published:** 2026-01-29

**Authors:** Ryosuke Uekusa, Akira Yokoi, Eri Watanabe, Mikako Inoue, Katsuhiko Mizuno, Kosuke Yoshida, Nobuhisa Yoshikawa, Kaoru Niimi, Shiro Suzuki, Hiroaki Kajiyama

**Affiliations:** 1 Department of Obstetrics and Gynecology, Nagoya University Graduate School of Medicine, Nagoya, Japan; 2 Department of Gynecologic Oncology, Aichi Cancer Center Hospital, Nagoya, Japan; 3 Institute for Advanced Research, Nagoya University, Nagoya, Japan

**Keywords:** myelodysplastic syndrome, ovarian cancer, PARP inhibitors, real-world data, therapy-related myeloid neoplasms

## Abstract

**Background:**

Poly(ADP-ribose) polymerase inhibitors (PARPi) have significantly improved outcomes in ovarian cancer. However, therapy-related myeloid neoplasms (t-MNs) have emerged as rare but serious late complications. Although an increased incidence of t-MNs has been reported following PARPi exposure, clinical predictors remain poorly understood.

**Methods:**

This retrospective study analyzed 181 patients with ovarian cancer treated with PARPi at two Japanese institutions. Clinical characteristics and routine hematologic parameters were compared between patients with and without t-MNs. Hematological values were assessed at initial diagnosis, PARPi initiation, 4 weeks after initiation, and at nadir within 12 weeks. Relative changes from initial diagnosis and from PARPi initiation to nadir were also evaluated.

**Results:**

t-MNs developed in 6 (3.3%) patients. All patients in the t-MN group had received multiple platinum-containing regimens, and in five of the six cases, t-MN was diagnosed more than 5 years after initial diagnosis. No definitive clinical predictors were identified, although the t-MN group tended to have higher body mass index. Median white blood cell (WBC), hemoglobin, and platelet counts, as well as their relative changes from initial diagnosis or PARPi initiation to nadir, did not differ significantly between the groups. However, the t-MN group exhibited a larger reduction in WBC count from PARPi initiation to nadir, not statistically significant.

**Conclusions:**

This real-world study highlights the importance of survivorship care in the era of improved outcomes for ovarian cancer. Continued long-term hematologic monitoring, along with the collection of more cases, is essential to elucidate risk factors for t-MNs in patients receiving PARPi therapy.

## 1 Introduction

Ovarian cancer is among the most lethal gynecologic malignancies worldwide (1), with high-grade serous ovarian carcinoma (HGSC) accounting for the majority of advanced cases (2). Despite advances in cytoreductive surgery and platinum-based chemotherapy, most patients eventually relapse, underscoring the urgent need for effective maintenance strategies (3, 4). The advent of poly(ADP-ribose) polymerase inhibitors (PARPi) has transformed ovarian cancer therapy, particularly in patients harboring *BRCA* mutations or broader homologous recombination deficiency (HRD) (5–7). As a result, PARPi have become essential agents in maintenance therapy, contributing to prolonged survival.

Therapy-related myeloid neoplasms (t-MNs)—including therapy-related acute myeloid leukemia (t-AML), myelodysplastic syndromes (t-MDS), and myelodysplastic/myeloproliferative neoplasms (t-MDS/MPN)—have long been recognized as serious late complications with a poor prognosis, with a 5-year survival of approximately 10% (8–10). Historically, alkylating agents and topoisomerase II inhibitors have been implicated in t-MN development (11, 12). Patients with ovarian cancer are at increased risk because of repeated platinum exposure and extended survival (8, 13).

Although PARPi have markedly improved outcomes, they also raise concerns regarding therapy-related complications. With longer survival durations, therapy-related late complications have gained attention. Recent analyses have reported two- to four-fold higher odds of t-MN development in patients treated with PARPi compared with placebo (14–16). Given the incorporation of PARPi into both frontline and recurrent settings, assessing the risk of t-MNs in ovarian cancer has become crucial. Real-world data are needed to capture long-term hematologic toxicities that may not be fully represented in clinical trials.

In this context, we retrospectively analyzed 181 patients with ovarian cancer treated with PARPi. This study aimed to determine whether clinical features and routine laboratory parameters could predict the onset of t-MNs. Although no definitive predictors were identified, our findings emphasize the increasing incidence of t-MNs in real-world practice and highlight the pressing need for predictive strategies to optimize the long-term management of patients receiving PARPi.

## 2 Methods

This study retrospectively reviewed the medical records of 181 patients with ovarian cancer who received olaparib and/or niraparib at Nagoya University Hospital and Aichi Cancer Center Hospital (Nagoya, Japan) between May 2018 and December 2022. Eligible patients received maintenance therapy for advanced epithelial ovarian, fallopian tube, or primary peritoneal cancer following first-line platinum-based chemotherapy, or for recurrent disease following platinum-based chemotherapy. Clinical data collected included patient background (age, body mass index [BMI], smoking, and alcohol consumption history), histological subtype, germline *BRCA* mutation, and homologous recombination (HR) status, prior chemotherapy regimens, adverse events, and serial blood test results.

This study was approved by the Ethics Committees of Nagoya University and Aichi Cancer Center (Approval No. 2013-0078). All procedures were conducted in accordance with institutional guidelines and the principal of the Declaration of Helsinki. Written informed consent was obtained from all participants.

Statistical analyses were performed using GraphPad Prism version 10. The Mann–Whitney U test or chi-square test was applied for between-group comparisons, and a p-value <0.05 was considered statistically significant.

## 3 Results

### 3.1 Patient characteristics and treatment history

Among the 181 patients, 6 developed t-MNs. Patient characteristics are summarized in **Table 1**. All six patients received olaparib therapy, and one patient also received niraparib. The t-MN and non-t-MN groups included 6 and 175 patients, respectively. The median age was 55 years (range, 39–66) in the t-MN group and 59 years (range, 23–80) in the non-t-MN group. Median BMI was 23.4 (range, 21.8–25.9) and 21.3 (range, 14.2–32.8) kg/m^2^, respectively. The t-MN group tended to have higher BMI values; however, no significant differences were observed in any clinical factors. No significant differences were found in the histological subtype, genetic profiles, and PARPi type between the two groups (**Table 1**).

**Table 1:** Patient characteristics.

Variable	Category	t-MN (N = 6)	non-t-MN (N = 175)	*p* value
Age		55 (39–66)	59 (23–80)	0.57
BMI		23.4 (21.8–25.9)	21.3 (14.2–32.8)	0.11
Smoking		1 (16.7%)	12 (11.4%)	0.53
Drinking		0 (0.0%)	19 (10.9%)	>0.99
Diabetes		0 (0.0%)	14 (8.0%)	>0.99
Histologic type				0.72
	Serous	5 (83.3%)	142 (81.1%)	
	Endometrioid	1 (16.7%)	14 (8.0%)	
	Clear	0 (0.0%)	10 (5.7%)	
	Carcinosarcoma	0 (0.0%)	1 (0.6%)	
	Unknown	0 (0.0%)	8 (4.6%)	
g*BRCA* status				>0.99
	Positive	1 (16.7%)	27 (15.3%)	
	Negative	2 (33.3%)	69 (34.3%)	
	Unknown	3 (50.0%)	88 (50.3%)	
HR status				0.51
	Deficient	0 (0.0%)	23 (13.1%)	
	Proficient	1 (16.7%)	13 (7.4%)	
	Unknown	5 (83.3%)	139 (79.4%)	
PARPi				0.44
	Olaparib	6(100.0%)	126 (78.0%)	
	Niraparib	1(16.7%)	49 (28.0%)	

**Figure 1:**
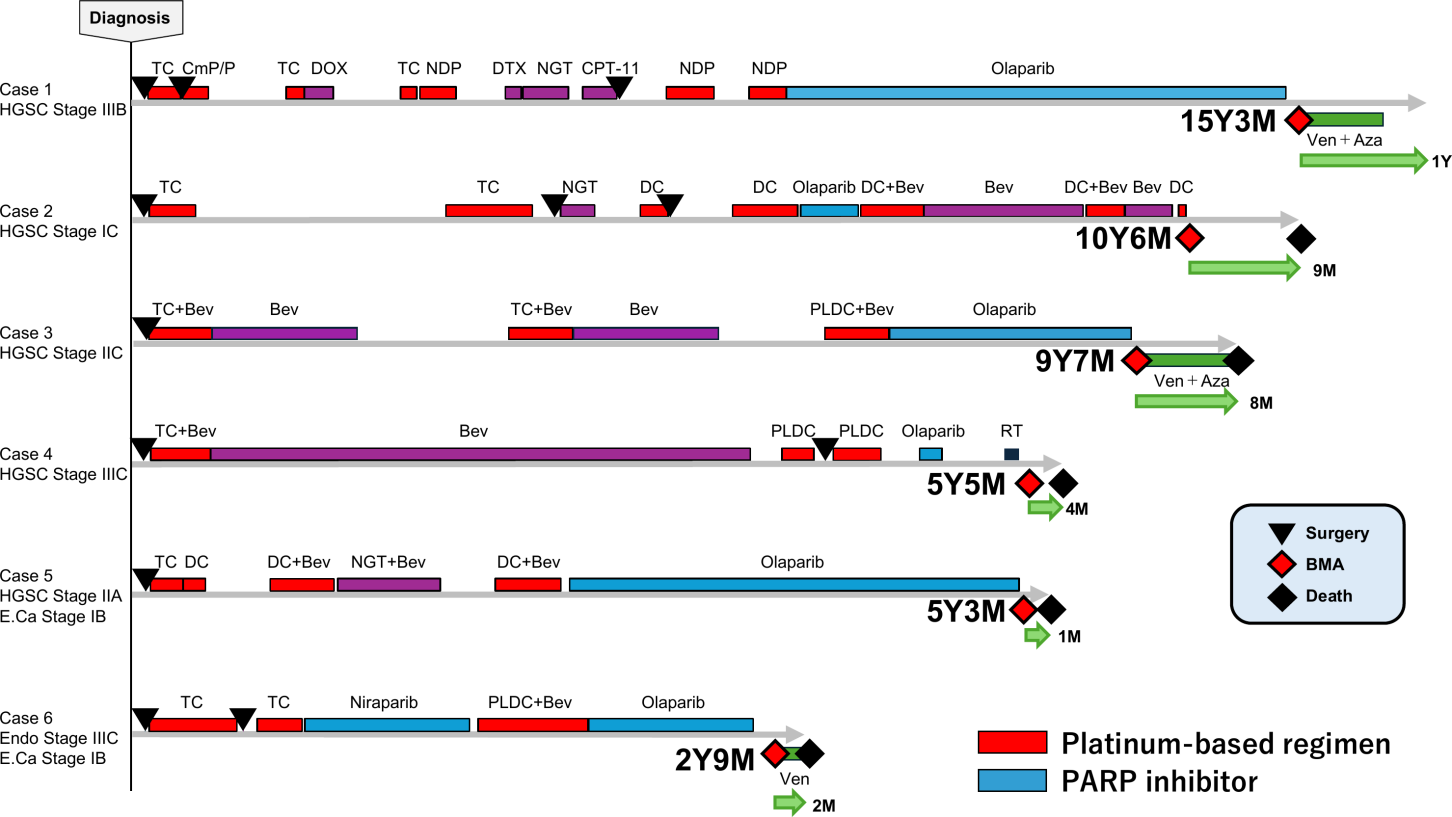
Clinical courses of six patients who developed t-MNs following PARPi therapy. Each bar represents the treatment timeline for a patient, beginning at the initial diagnosis. The red, blue, green, and purple segments indicate platinum-based regimens, PARPi therapy, regimens administered for t-MN, and other treatments, respectively. Abbreviations: HGSC, high-grade serous carcinoma; E.Ca, endometrial carcinoma; Endo, endometrioid carcinoma; TC, paclitaxel + carboplatin; CmP/P, cyclophosphamide + cisplatin + peplomycin; DOX, doxorubicin; NDP, nedaplatin; DTX, docetaxel; NGT, nogitecan; CPT-11, irinotecan; Ven, venetoclax; AZA, azacitidine; DC, docetaxel + carboplatin; Bev, bevacizumab; RT, radiation therapy; BMA, bone marrow aspiration.

**Figure 1** summarizes the clinical courses of the six cases. Five patients achieved long-term survival, living >5 years after treatment initiation. All six patients who subsequently developed t-MN received multiple platinum-based regimens. Following t-MN onset, treatment outcomes were generally poor, and five of the six patients died within 1 year, consistent with the poor prognosis previously reported. Brief clinical courses of each case are provided below. Cytogenetic analyses demonstrated a very poor karyotype in all evaluable t-MN cases. All patients were classified as very high risk according to the IPSS-R. As shown in **Figure 1**, the timelines clearly illustrate the duration of PARPi exposure and the latency to t-MN development in each case.

#### 3.1.1 Case 1

A 49-year-old woman was diagnosed with stage IIIB ovarian cancer, histology high-grade serous carcinoma (HGSC). Germline *BRCA* and HRD testing were not performed. She underwent interval debulking surgery (IDS) following neoadjuvant chemotherapy (NAC). After multiple lines of chemotherapy for pelvic recurrence, secondary debulking surgery (SDS) was performed 7 years after the initial treatment. Olaparib was subsequently initiated as maintenance therapy for platinum-sensitive relapse. She received 12 cycles of TC (paclitaxel plus carboplatin), 3 of CmP/P (cyclophosphamide, cisplatin, and peplomycin), and 15 of nedaplatin. She continued olaparib for 5 years and 1 month. At 15 years and 3 months after the initial treatment, peripheral blood blasts were detected. Bone marrow aspiration revealed normocellular marrow (**Supplementary Figure S1a**) with dysplastic changes, including Pelger–Huët-like nuclear abnormalities (**Supplementary Figure S1b**), neutrophil hypogranularity (**Supplementary Figure S1c**), and micromegakaryocytes (**Supplementary Figure S1d**). Myeloblasts with fine chromatin and prominent nucleoli comprised 18.5%, consistent with myelodysplastic syndrome with excess blasts-2 (MDS-EB2). Her condition subsequently progressed to AML, and she was treated with venetoclax plus azacitidine. She remained alive 1 year after the diagnosis of t-MN.

#### 3.1.2 Case 2

A 48-year-old woman was diagnosed with stage IC ovarian cancer, histology HGSC. Germline *BRCA* and HRD testing were not performed. She underwent primary debulking surgery (PDS) followed by TC. Pelvic peritoneal recurrence was managed with chemotherapy and surgery. Olaparib was initiated as maintenance therapy for platinum-sensitive relapse but discontinued after 5 months because of disease progression. After further chemotherapy, blasts appeared in the peripheral blood 10 years and 6 months after the initial treatment. Bone marrow aspiration showed hypercellular marrow (**Supplementary Figure S1e**) with micromegakaryocytes, pseudo-Pelger–Huët anomalies, granulocytic hypogranularity, and megaloblastic erythroid changes (**Supplementary Figure S1f**). Large to giant platelets were also observed (**Supplementary Figure S1g**). Myeloblasts accounted for 5.0%, leading to a diagnosis of MDS-EB1. She received 16 TC cycles and 18 DC (docetaxel plus carboplatin) cycles, and took olaparib for 5 months. She died of disease progression 8 months after the MDS diagnosis.

#### 3.1.3 Case 3

A 39-year-old woman was diagnosed with stage IIC ovarian cancer, histology HGSC. Germline *BRCA* and HRD testing were not performed. She underwent PDS and received TC plus bevacizumab. After recurrence at the vaginal cuff, she received chemotherapy. Olaparib was initiated as maintenance therapy for platinum-sensitive relapse. At 9 years and 7 months after the initial treatment, she developed MDS. She received a total of 12 TC cycles and 6 PLD-C cycles (pegylated liposomal doxorubicin plus carboplatin) and took olaparib for 3 years and 3 months. Venetoclax plus azacitidine was started for AML transformation, but she died of disease progression 8 months after the diagnosis of t-MN.

#### 3.1.4 Case 4

A 66-year-old woman was diagnosed with stage IIIC ovarian cancer, with HGSC histology, and a germline *BRCA* mutation. HRD testing was not performed. She underwent PDS followed by TC plus bevacizumab. After chemotherapy for pelvic dissemination, SDS was performed. Additional chemotherapy was administered, and olaparib was started but discontinued after 2 months because of persistent myelosuppression. Radiotherapy was performed for pelvic nodal recurrence. At 5 years and 5 months after the initial treatment, she was diagnosed with MDS. She had received six TC cycles and six PLD-C cycles and was treated with olaparib for 2 months. She died of disease progression 3 months after the MDS diagnosis.

#### 3.1.5 Case 5

A 63-year-old woman presented with synchronous double cancers: stage IIA ovarian cancer (HGSC) and stage IB endometrial cancer (endometrioid carcinoma). She was positive for a germline *BRCA* mutation and did not undergo HRD testing. She underwent PDS and then received adjuvant chemotherapy. Multiple cycles of chemotherapy were given for pelvic recurrence, and olaparib was started as maintenance for platinum-sensitive relapse. After 2 years and 3 months of olaparib, peripheral blood blasts appeared. She was diagnosed with MDS 5 years and 3 months after the initial treatment. She had received 3 TC cycles and 13 PLD-C cycles, and continued on olaparib for 2 years and 3 months. She died of AML transformation 1 month after the t-MN diagnosis.

#### 3.1.6 Case 6

A 61-year-old woman had synchronous double cancers: stage IIIC ovarian cancer and stage IA endometrial cancer, both endometrioid carcinomas. She was negative for germline *BRCA*, and her HR status was proficient. She underwent IDS following NAC. Maintenance therapy with niraparib was initiated. Olaparib was later administered after chemotherapy for disseminated recurrence. At 2 years and 9 months after the initial treatment, she was diagnosed with MDS. She had received 9 TC cycles and 6 PLD-C cycles, and was treated with niraparib for 8 months and olaparib for 8 months. Venetoclax was administered, but she died of ovarian cancer progression 2 months after the t-MN diagnosis.

### 3.2 Hematologic parameters at baseline and during PARPi therapy

**Figure 2:**
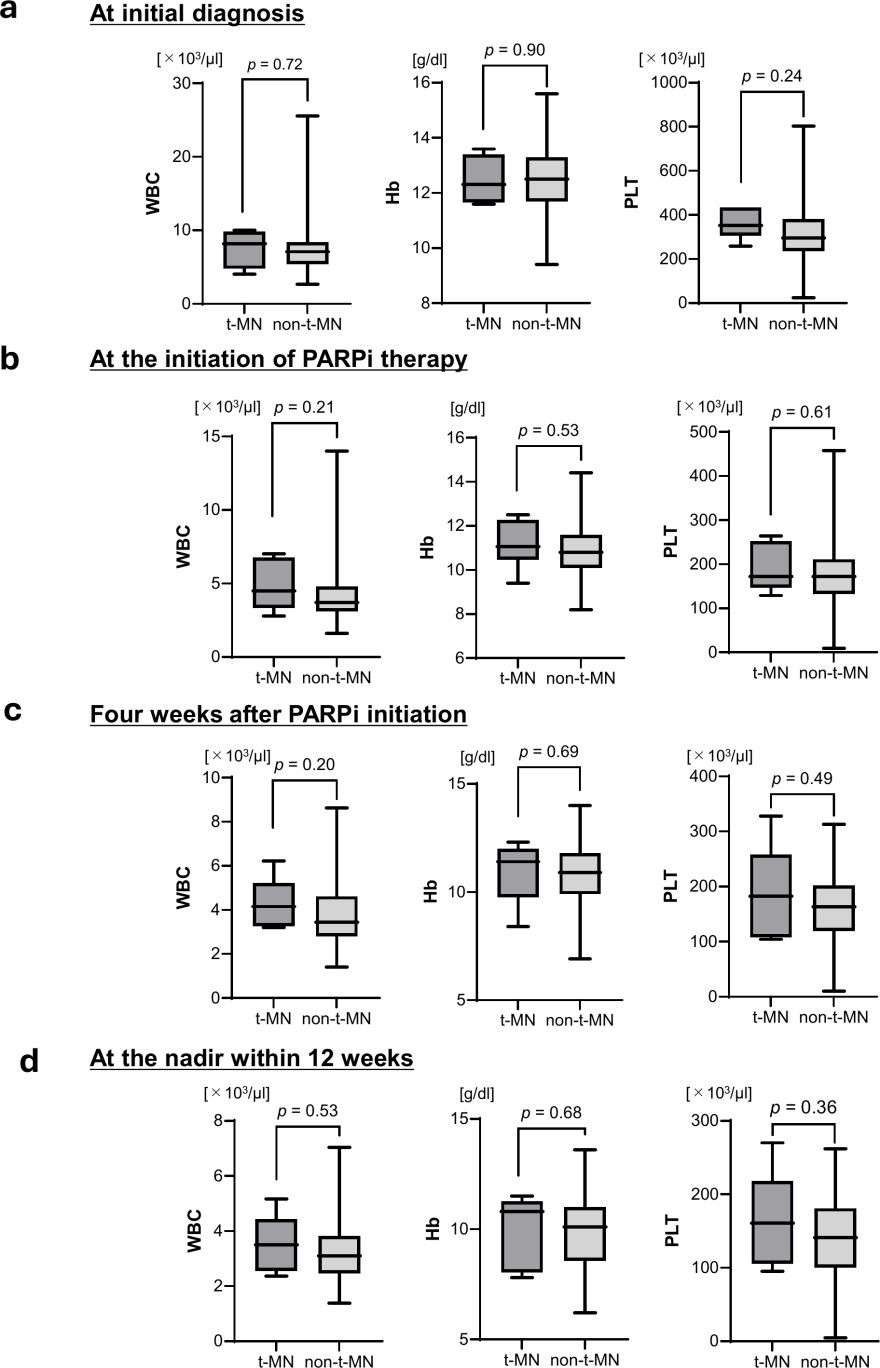
Hematological parameters at baseline and during PARPi therapy. **(a)** At initial diagnosis, no significant differences were noted in WBC count (median 8.2 vs. 7.1 × 10³/µL), hemoglobin (12.3 vs. 12.5 g/dL), or platelet count (352 vs. 296.5 × 10³/µL) between patients who developed and those who did not develop t-MN. **(b)** At the initiation of PARPi therapy, the medians were 4.5 vs. 3.7 × 10³/µL for WBC, 11.1 vs. 10.8 g/dL for hemoglobin, and 172 vs. 172 × 10³/µL for platelets, with no significant differences. **(c)** Four weeks after PARPi initiation, the values were 4.2 vs. 3.4 × 10³/µL, 11.4 vs. 10.9 g/dL, and 182 vs. 163 × 10³/µL, respectively, again showing no significant differences. **(d)** At the nadir within 12 weeks, the values were 3.5 vs. 3.1 × 10³/µL, 10.8 vs. 10.1 g/dL, and 161 vs. 141 × 10³/µL, respectively, with no significant differences between the two groups. Abbreviations: PARPi, poly(ADP-ribose) polymerase; WBC, white blood cells.

This study examined hematological parameters not only at the time of initial diagnosis and the initiation of PARPi treatment, but also during PARPi therapy (4 weeks after initiation and at nadir within 12 weeks) to determine whether these routine blood counts could predict subsequent t-MN development. At the initial diagnosis (**Figure 2a**), the median WBC count, hemoglobin level, and platelet count were 8.2 vs. 7.1 × 10³/µL, 12.3 vs. 12.5 g/dL, and 352 vs. 296.5 × 10³/µL, respectively, with no significant differences between the t-MN and non-t-MN groups (all ns). At the initiation of PARPi therapy (**Figure 2b**), the corresponding median values were 4.5 vs. 3.7 × 10³/µL, 11.1 vs. 10.8 g/dL, and 172 vs. 172 × 10³/µL, respectively, again without significant differences (all ns). Four weeks after initiation (**Figure 2c**), the median values were 4.2 vs. 3.4 × 10³/µL, 11.4 vs. 10.9 g/dL, and 182 vs. 163 × 10³/µ, respectively, also without significant differences (all ns). Similarly, at the nadir within 12 weeks after PARPi initiation (**Figure 2d**), the median values were 3.5 vs. 3.1 × 10³/µL, 10.8 vs. 10.1 g/dL, and 161 vs. 141 × 10³/µL, respectively, showing no significant differences (all ns).

### 3.3 Changes in hematological parameters

**Figure 3:**
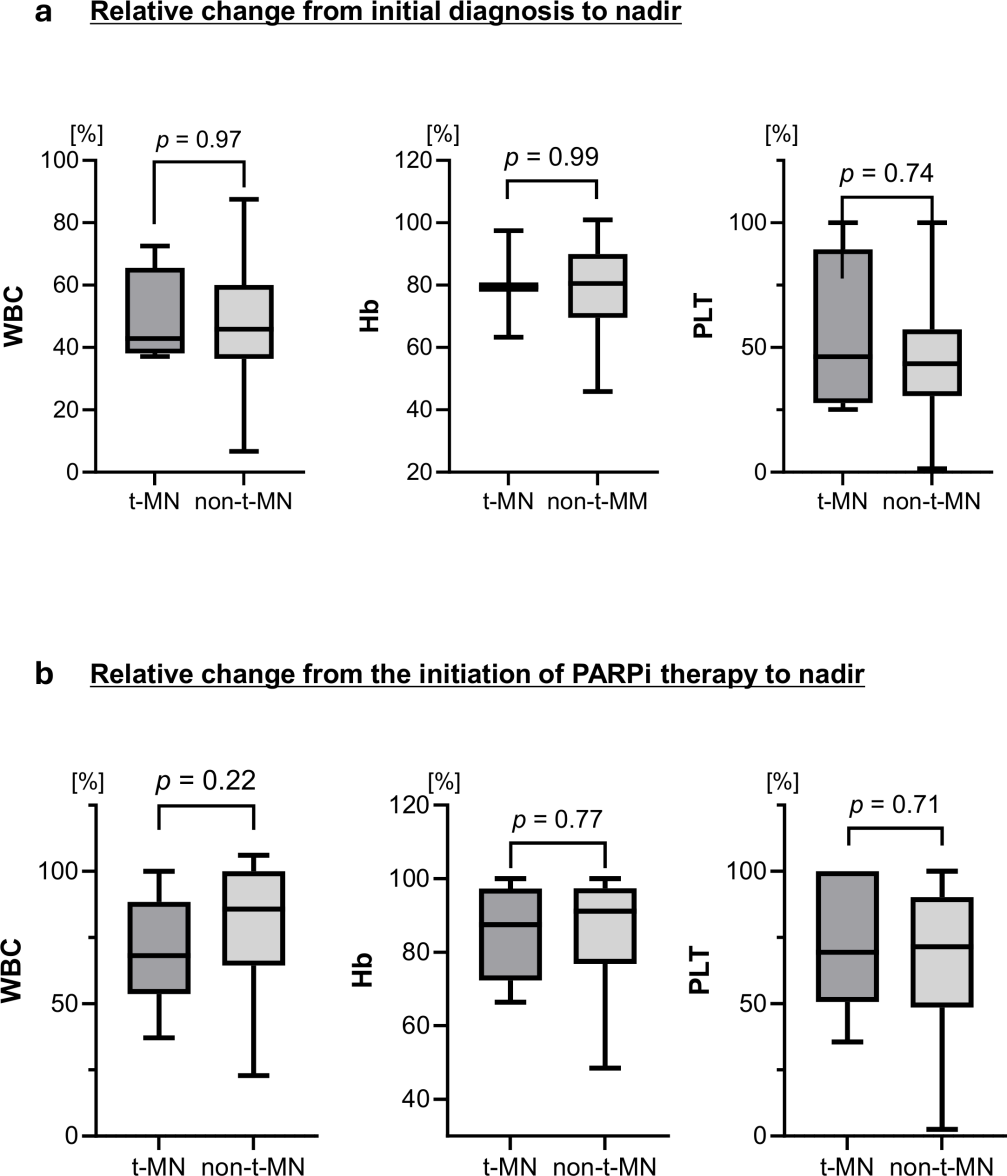
Changes in hematological parameters during PARPi therapy. **(a)** Relative change from initial diagnosis to nadir: the median rates were 42.8% vs. 45.9% for WBC, 79.4% vs. 80.6% for hemoglobin, and 46.3% vs. 43.5% for platelets, with no significant differences. **(b)** Relative change from the initiation of PARPi therapy to nadir: the median rates were 68.2% vs. 85.8% for WBC, 87.5% vs. 91.2% for hemoglobin, and 69.5% vs. 71.5% for platelets, again showing no significant differences between the two groups. Abbreviations: PARPi, poly(ADP-ribose) polymerase; WBC, white blood cells.

To further explore the potential association between the degree of bone marrow suppression following PARPi therapy and subsequent t-MN onset, ratios of nadir values within 12 weeks were compared with those at the initial diagnosis and at the start of PARPi therapy. The degree of hematological suppression, expressed as the rate of change, is shown in **Figure 3**. From the initial diagnosis to the nadir (**Figure 3a**), the median rates of change were 42.8% vs. 45.9% for WBC, 79.4% vs. 80.6% for hemoglobin, and 46.3% vs. 43.5% for platelets, with no significant differences (all ns). From the initiation of PARPi treatment to the nadir within 12 weeks (**Figure 3b**), the median values were 68.2% vs. 85.8%, 87.5% vs. 91.2%, and 69.5% vs. 71.5%, respectively, again showing no significant differences (all ns).

## 4 Discussion

In this retrospective analysis of 181 patients with ovarian cancer treated with PARPi, no definitive clinical predictors of t-MNs were identified. However, preliminary trends suggested that the t-MN group tended to have higher BMI and greater WBC count reduction from PARPi initiation to nadir. Although these findings did not reach statistical significance–likely due to the small number of cases–they may represent possible features warranting validation in larger cohorts.

The incidence of t-MN (3.3%) in the present study was slightly higher than that reported in randomized controlled trials (RCTs) of PARPi (9). In the NOVA, SOLO-1, and PRIMA trials, the incidence of MDS/AML ranged from 0.5% to 1.5% with longer-term follow-up (7, 17, 18). Several factors may explain this discrepancy between RCTs and real-world data. Trial participants are generally younger, have fewer comorbidities, and receive less prior treatment exposure, whereas real-world populations often include patients with extensive cumulative platinum exposure, multiple prior regimens, and older age—all recognized as risk factors for t-MNs (19, 20). Furthermore, the survival benefit conferred by PARPi has extended progression-free intervals, resulting in a growing population of long-term survivors who live long enough to develop late toxicities. Previous studies have shown that t-MNs typically emerge at a median of approximately 5 years after initial treatment (8), consistent with our observation that most t-MN cases developed long after the initiation of ovarian cancer therapy. Together, these findings emphasize the importance of survivorship care and long-term hematologic surveillance in patients with ovarian cancer.

The pathogenesis of t-MN is complex and multifactorial, involving DNA repair gene mutations (e.g., BRCA1/2), DNA damage induced by chemotherapy or radiation therapy, bone marrow microenvironment injury, and clonal hematopoiesis of indeterminate potential (CHIP) (8, 9, 21–23). Historically, alkylating agents and topoisomerase II inhibitors were strongly implicated, with latency periods of 5–10 and 1–5 years, respectively (11, 19). In the PARPi era, additional mechanisms may contribute. PARPi inhibit base excision repair, inducing synthetic lethality in *BRCA*-mutant or HRD-deficient tumor cells but potentially also impairing DNA repair in normal hematopoietic stem cells (10). In patients with germline *BRCA* mutations, tumor and hematopoietic compartments likely exhibit HRD, potentially increasing susceptibility to therapy-induced mutagenesis under PARPi exposure. Moreover, recent evidence highlights CHIP as a critical predisposition for t-MNs. CHIP-related mutations, particularly in *TP53*, *DNMT3A*, *TET2*, and *RUNX1*, can precede overt leukemia by years (21, 23–25). Chemotherapy, radiotherapy, and PARPi may apply selective pressure, promoting expansion of these mutant clones (15, 26). Although next-generation sequencing can detect CHIP, routine monitoring is impractical. Thus, accessible predictors—such as hematologic parameters—remain a critical unmet need. Although CHIP profiling was not performed in this study, occult CHIP mutations may have contributed to some cases, highlighting an important direction for future translational research. Although PARPi-induced clonal expansions, including *TP53*-mutant clones, have been reported to regress after treatment cessation (27), our observation that two patients developed t-MN after PARPi discontinuation suggests that clonal regression does not necessarily eliminate subsequent risk. In these cases, additional cytotoxic chemotherapy or radiotherapy may have contributed further DNA damage and selective pressure, promoting clonal evolution toward t-MN.

This study has several strengths. To our knowledge, it represents one of the largest real-world analyses addressing the association between PARPi and t-MN development in ovarian cancer. Unlike clinical trial populations, our dataset reflects an unselected patient group treated in routine practice. Nevertheless, several limitations should be acknowledged. The small number of patients with t-MNs limited the study’s statistical power. Additionally, molecular profiling, including CHIP-related mutation analysis, was not performed, which limited the investigation of the pathogenetic mechanism. Follow-up duration may also have been insufficient to capture late-onset cases, as latency can exceed a decade (11, 19). Clinically, a major challenge will be balancing the benefits of PARPi against the rare but serious risk of t-MNs. This underscores the difficulty in making decisions by both clinicians and patients and highlights the need for strategies that enable risk stratification before treatment. Ideally, pretreatment risk assessment tools would enable individualized patient selection to maximize benefit while minimizing severe late toxicities. Collaborative multicenter studies with larger cohorts and integrated genomic analyses are essential to establish definitive risk factors.

## 5 Conclusion

Although no definitive predictors of t-MN were identified, the findings underscore the growing importance of long-term survivorship management in ovarian cancer. The combination of improved survival, greater treatment exposure, and pre-existing clonal hematopoiesis may contribute to the increasing incidence of t-MN in this population. Future investigations and accumulation of clinical cases are necessary to identify risk factors for t-MNs in patients treated with PARPi.

## Data Availability

The datasets presented in this article are not readily available because they contain sensitive patient information. Requests to access the datasets should be directed to the corresponding author.

## References

[1] Bray F Laversanne M Sung H Ferlay J Siegel RL Soerjomataram I . Global cancer statistics 2022: GLOBOCAN estimates of incidence and mortality worldwide for 36 cancers in 185 countries. CA Cancer J Clin. (2024) 74:229–63. doi: 10.3322/caac.21834, PMID: 38572751

[2] Pavlidis N Rassy E Vermorken JB Assi T Kattan J Boussios S . The outcome of patients with serous papillary peritoneal cancer, fallopian tube cancer, and epithelial ovarian cancer by treatment eras: 27 years data from the SEER registry. Cancer Epidemiol. (2021) 75:102045. doi: 10.1016/j.canep.2021.102045, PMID: 34638085

[3] Kurnit KC Fleming GF Lengyel E . Updates and new options in advanced epithelial ovarian cancer treatment. Obstet Gynecol. (2021) 137:108–21. doi: 10.1097/AOG.0000000000004173, PMID: 33278287 PMC7737875

[4] Pignata S CC S Du Bois A Harter P Heitz F . Treatment of recurrent ovarian cancer. Ann Oncol. (2017) 28:viii51–viii6. doi: 10.1093/annonc/mdx441, PMID: 29232464

[5] Pujade-Lauraine E Ledermann JA Selle F Gebski V Penson RT Oza AM . Olaparib tablets as maintenance therapy in patients with platinum-sensitive, relapsed ovarian cancer and a BRCA1/2 mutation (SOLO2/ENGOT-Ov21): a double-blind, randomised, placebo-controlled, phase 3 trial. Lancet Oncol. (2017) 18:1274–84. doi: 10.1016/S1470-2045(17)30469-2, PMID: 28754483

[6] Ray-Coquard I Pautier P Pignata S Perol D Gonzalez-Martin A Berger R . Olaparib plus bevacizumab as first-line maintenance in ovarian cancer. N Engl J Med. (2019) 381:2416–28. doi: 10.1056/NEJMoa1911361, PMID: 31851799

[7] Moore K Colombo N Scambia G Kim BG Oaknin A Friedlander M . Maintenance olaparib in patients with newly diagnosed advanced ovarian cancer. N Engl J Med. (2018) 379:2495–505. doi: 10.1056/NEJMoa1810858, PMID: 30345884

[8] Morton LM Dores GM Schonfeld SJ Linet MS Sigel BS Lam CJK . Association of chemotherapy for solid tumors with development of therapy-related myelodysplastic syndrome or acute myeloid leukemia in the modern era. JAMA Oncol. (2019) 5:318–25. doi: 10.1001/jamaoncol.2018.5625, PMID: 30570657 PMC6439835

[9] Morice PM Leary A Dolladille C Chretien B Poulain L Gonzalez-Martin A . Myelodysplastic syndrome and acute myeloid leukaemia in patients treated with PARP inhibitors: a safety meta-analysis of randomised controlled trials and a retrospective study of the WHO pharmacovigilance database. Lancet Haematol. (2021) 8:e122–e34. doi: 10.1016/S2352-3026(20)30360-4, PMID: 33347814

[10] McNerney ME Godley LA Le Beau MM . Therapy-related myeloid neoplasms: when genetics and environment collide. Nat Rev Cancer. (2017) 17:513–27. doi: 10.1038/nrc.2017.60, PMID: 28835720 PMC5946699

[11] Godley LA Larson RA . Therapy-related myeloid leukemia. Semin Oncol. (2008) 35:418–29. doi: 10.1053/j.seminoncol.2008.04.012, PMID: 18692692 PMC2600445

[12] Smith SM Le Beau MM Huo D Karrison T Sobecks RM Anastasi J . Clinical-cytogenetic associations in 306 patients with therapy-related myelodysplasia and myeloid leukemia: the University of Chicago series. Blood. (2003) 102:43–52. doi: 10.1182/blood-2002-11-3343, PMID: 12623843

[13] Sarkar J Chakraborti T Chowdhury A Bhuyan R Chakraborti S . Protective role of epigallocatechin-3-gallate in NADPH oxidase-MMP2-Spm-Cer-S1P signalling axis mediated ET-1 induced pulmonary artery smooth muscle cell proliferation. J Cell Commun Signal. (2019) 13:473–89. doi: 10.1007/s12079-018-00501-7, PMID: 30661173 PMC6946791

[14] Travaglini S Marinoni M Visconte V Guarnera L . Therapy-related myeloid neoplasm: biology and mechanistic aspects of Malignant progression. Biomedicines. (2024) 12. doi: 10.3390/biomedicines12051054, PMID: 38791019 PMC11118122

[15] Kwan TT Oza AM Tinker AV Ray-Coquard I Oaknin A Aghajanian C . Preexisting TP53-variant clonal hematopoiesis and risk of secondary myeloid neoplasms in patients with high-grade ovarian cancer treated with rucaparib. JAMA Oncol. (2021) 7:1772–81. doi: 10.1001/jamaoncol.2021.4664, PMID: 34647981 PMC8517887

[16] Mirza MR Avall Lundqvist E Birrer MJ dePont Christensen R Nyvang GB Malander S . Niraparib plus bevacizumab versus niraparib alone for platinum-sensitive recurrent ovarian cancer (NSGO-AVANOVA2/ENGOT-ov24): a randomised, phase 2, superiority trial. Lancet Oncol. (2019) 20:1409–19. doi: 10.1016/S1470-2045(19)30515-7, PMID: 31474354

[17] Mirza MR Monk BJ Herrstedt J Oza AM Mahner S Redondo A . Niraparib maintenance therapy in platinum-sensitive, recurrent ovarian cancer. N Engl J Med. (2016) 375:2154–64. doi: 10.1056/NEJMoa1611310, PMID: 27717299

[18] Gonzalez-Martin A Pothuri B Vergote I DePont Christensen R Graybill W Mirza MR . Niraparib in patients with newly diagnosed advanced ovarian cancer. N Engl J Med. (2019) 381:2391–402. doi: 10.1056/NEJMoa1910962, PMID: 31562799

[19] Leone G Pagano L Ben-Yehuda D Voso MT . Therapy-related leukemia and myelodysplasia: susceptibility and incidence. Haematologica. (2007) 92:1389–98. doi: 10.3324/haematol.11034, PMID: 17768113

[20] Bhatia S . Therapy-related myelodysplasia and acute myeloid leukemia. Semin Oncol. (2013) 40:666–75. doi: 10.1053/j.seminoncol.2013.09.013, PMID: 24331189 PMC3867743

[21] Gillis NK Ball M Zhang Q Ma Z Zhao Y Yoder SJ . Clonal haemopoiesis and therapy-related myeloid Malignancies in elderly patients: a proof-of-concept, case-control study. Lancet Oncol. (2017) 18:112–21. doi: 10.1016/S1470-2045(16)30627-1, PMID: 27927582 PMC7771361

[22] Bernard E Nannya Y Hasserjian RP Devlin SM Tuechler H Medina-Martinez JS . Implications of TP53 allelic state for genome stability, clinical presentation and outcomes in myelodysplastic syndromes. Nat Med. (2020) 26:1549–56. doi: 10.1038/s41591-020-1008-z, PMID: 32747829 PMC8381722

[23] Wong TN Ramsingh G Young AL Miller CA Touma W Welch JS . Role of TP53 mutations in the origin and evolution of therapy-related acute myeloid leukaemia. Nature. (2015) 518:552–5. doi: 10.1038/nature13968, PMID: 25487151 PMC4403236

[24] Jaiswal S Fontanillas P Flannick J Manning A Grauman PV Mar BG . Age-related clonal hematopoiesis associated with adverse outcomes. N Engl J Med. (2014) 371:2488–98. doi: 10.1056/NEJMoa1408617, PMID: 25426837 PMC4306669

[25] Takahashi K Wang F Kantarjian H Doss D Khanna K Thompson E . Preleukaemic clonal haemopoiesis and risk of therapy-related myeloid neoplasms: a case-control study. Lancet Oncol. (2017) 18:100–11. doi: 10.1016/S1470-2045(16)30626-X, PMID: 27923552 PMC5405697

[26] Bolton KL Ptashkin RN Gao T Braunstein L Devlin SM Kelly D . Cancer therapy shapes the fitness landscape of clonal hematopoiesis. Nat Genet. (2020) 52:1219–26. doi: 10.1038/s41588-020-00710-0, PMID: 33106634 PMC7891089

[27] Nuttall Musson E Miller RE Mansour MR Lockley M Ledermann JA Payne EM . Monitoring clone dynamics and reversibility in clonal haematopoiesis and myelodysplastic neoplasm associated with PARP inhibitor therapy-a role for early monitoring and intervention. Leukemia. (2024) 38:215–8. doi: 10.1038/s41375-023-02040-6, PMID: 37978317 PMC10776406

