# Supplementary material for: Real-world data-based assessment of therapy-related myeloid neoplasms after poly(ADP-ribose) polymerase inhibitor treatment in ovarian cancer
Source: Front Oncol. 2026 Jan 29;16:1728766. doi: 10.3389/fonc.2026.1728766 (PMC12894034; doi:10.3389/fonc.2026.1728766)
Supplement: Supplementary file 1 [file DataSheet1.docx]

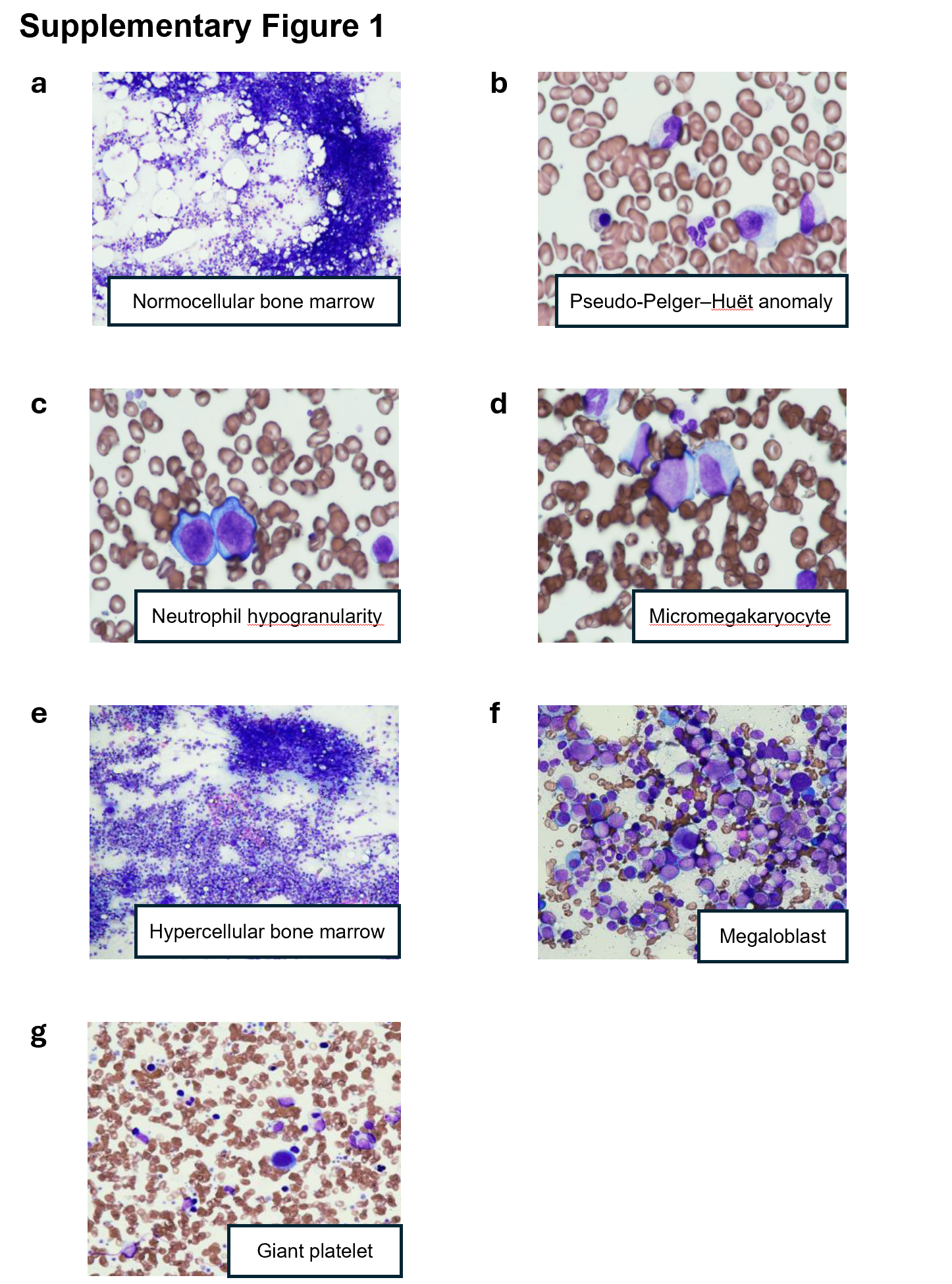


**Supplementary Figure 1. Representative bone marrow findings in patients with t-MN.**

(a–d) Findings from Case 1: (a) Normocellular bone marrow, (b) Pseudo–Pelger-Huët anomaly, (c) Neutrophil hypogranularity, and (d) Micromegakaryocyte. (e–g) Findings from Case 2: (e) Hypercellular bone marrow, (f) Megaloblastic erythroid changes, and (g) Giant platelets. These panels illustrate the dysplastic features observed in patients who developed t-MN after PARPi therapy.
